# Tirzepatide: a novel therapeutic approach for Alzheimer’s disease

**DOI:** 10.1007/s11011-025-01649-z

**Published:** 2025-06-11

**Authors:** Ghadah H. Alshehri, Hayder M. Al-kuraishy, Huda Jaber Waheed, Ali I. Al-Gareeb, Safaa A. Faheem, Athanasios Alexiou, Marios Papadakis, Gaber El-Saber Batiha

**Affiliations:** 1 Department of Pharmacy Practice, College of Pharmacy, Princess Nourah bint Abdulrahman University, Riyadh, Saudi Arabia; 2 Department of Clinical Pharmacology and Medicine, College of Medicine, Mustansiriyah University, Baghdad, Iraq; 3 Department Of Pharmacology and Toxicology, College of Pharmacy Mustansiriyah University, Baghdad, Iraq; 4 Department of Pharmacology and Toxicology, Faculty of Pharmacy, Egyptian Russian University, Cairo-Suez Road, Badr City, Cairo, 11829 Egypt; 5 Department of Research & Development, Funogen, Athens, 11741 Attiki Greece; 6 University Centre for Research & Development, Chandigarh University, Chandigarh-Ludhiana Highway, Mohali, Punjab India; 7 University Hospital Witten-Herdecke, University of Witten-Herdecke, Heusnerstrasse 40, 42283 Wuppertal, Germany; 8 Department of Pharmacology and Therapeutics, Faculty of Veterinary Medicine, Damanhour University, Damanhour, AlBeheira, 22511 Egypt

**Keywords:** Alzheimer's disease, Brain insulin signaling, Glucagon-like peptide one and gastric inhibitory polypeptide, Tirzepatide

## Abstract

Tirzepatide (TRZ) is a dual agonist of glucagon-like peptide 1 (GLP-1) and gastric inhibitory polypeptide (GIP) receptors that were recently approved for the treatment of type 2 diabetes (T2D) and obesity. Of note is that T2D and obesity, by inducing peripheral low-grade inflammation and oxidative stress, provoke the development of central neuroinflammation and oxidative stress. Together, T2D and obesity are regarded as potential risk factors implicated in the development and progression of Alzheimer’s disease (AD), which is the most common neurodegenerative disease and represents the most typical cause of dementia. Hence, targeting low-grade inflammation and oxidative stress in T2D and obesity by TRZ may reduce AD neuropathology. In addition, TRZ can inhibit the production of amyloid beta (Aβ) and associated neuroinflammation, oxidative stress, and neuronal apoptosis. However, the underlying neuroprotective mechanism of TRZ against AD is not entirely explained. Consequently, this mini-review aims to discuss the possible molecular mechanism of TRZ in AD.

## Introduction

**Fig. 1:**
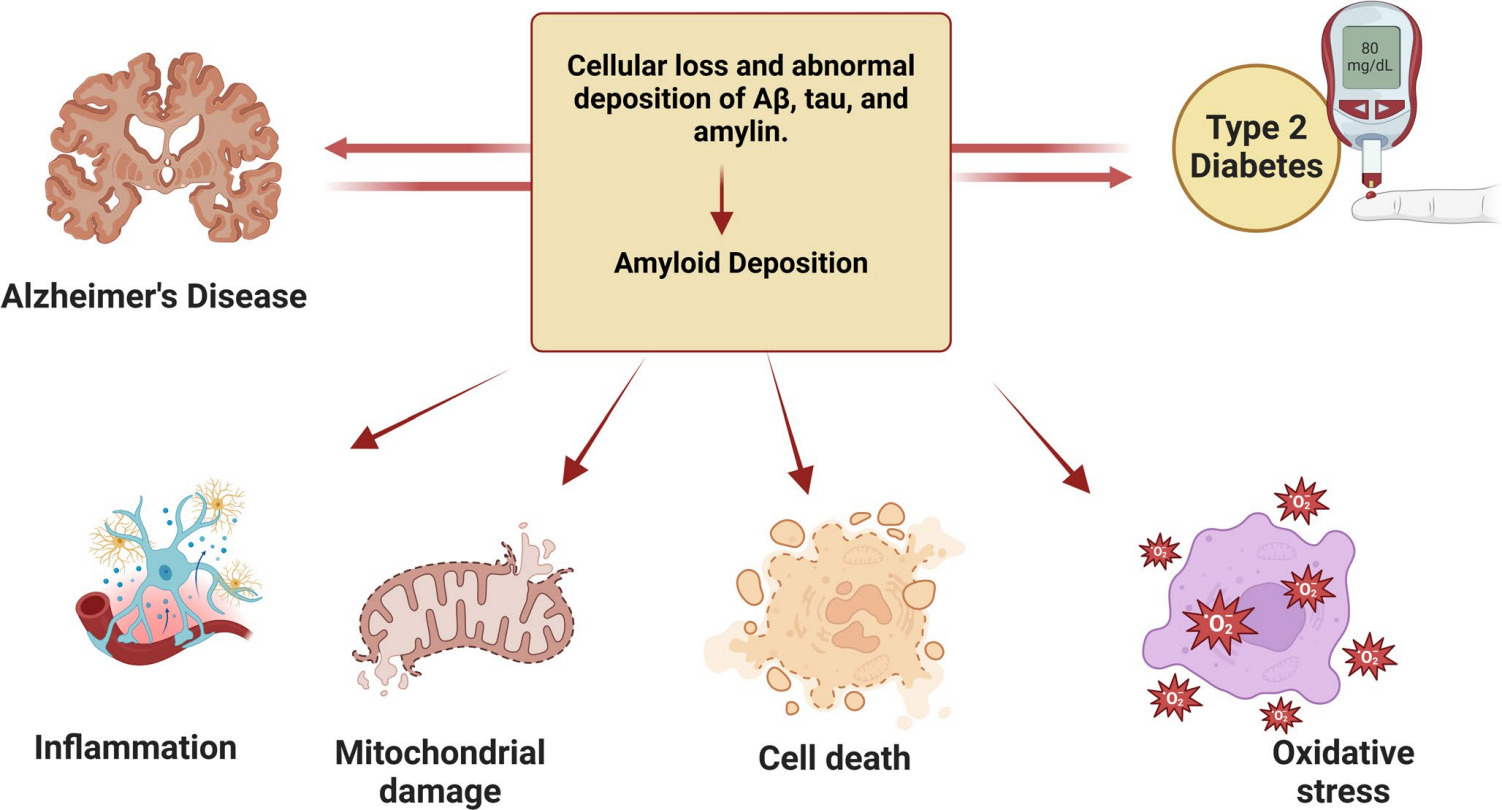
Mechanism of action of TRZ

Tirzepatide (TRZ) is an analog of the gastric inhibitory polypeptide (GIP) and acts as a dual agonist for glucagon-like peptide 1 (GLP-1) and GIP receptors (Rhea et al. 2024). Activation of GIP/GLP-1 results in systemic beneficial effects by modulating adipose tissue function and improvement of insulin sensitivity (Perovic et al. 2024) by inhibiting the release of glucagon hormone from pancreatic α cells and activating the release of insulin from pancreatic β cells (Forzano et al. 2022) (Fig. 1). TRZ activates GIP receptors more than GLP-1 receptors, leading to weight loss and activating the release of cytoprotective adiponectin (Willard et al. 2020). TRZ was approved in 2022 for the treatment of T2D and obesity, mainly in patients with cardio-metabolic disorders (Boutari et al. 2023). TRZ is more effective than GLP-1 receptor agonists such as dulaglutide in managing obesity and T2D (Powell and Taylor 2024). Furthermore, TRZ has diverse pleiotropic effects, such as anti-inflammatory, antioxidant, and anti-atherosclerotic effects, thereby decreasing cardiovascular complications (Taktaz et al. 2024).

TRZ is given by subcutaneous route only once per week, has a long half-life, and crosses BBB, leading to the induction of a satiety center in the hypothalamus (Boer et al. 2023). Extended use of TRZ is associated with the development of gastrointestinal adverse effects such as nausea, dyspepsia, vomiting, abdominal pain, diarrhea or constipation, and hypoglycemia. The rate for the development of adverse effects is correlated with the high dosage of TRZ (Jensen et al. 2024). The effective therapeutic dose of TRZ is 0.25 mg, though the toxic dose is > 10 mg. Therefore, TRZ has a good therapeutic window with minimal toxicity (Min and Bain 2021). TRZ is contraindicated in patients with multiple endocrine neoplasia syndrome type 2 and medullary thyroid cancer (Krauss et al. 2023).

Interestingly, GLP-1 and GIP receptors are widely expressed in the central nervous system (CNS), and insulin signaling regulates synaptic and cognitive functions (Zhang et al. 2023). It has been shown that dysregulation of the expression of GLP-1 and GIP receptors and impairment of insulin receptor substrate 1 (IRS-1) are implicated in the development and progression of neurodegenerative diseases such as Alzheimer’s disease (AD) (Talbot et al. 2012). Of note, AD-related pathologies prompt the activation of microglia and the release of pro-inflammatory/inflammatory cytokines, which inhibit insulin receptor substrate 1 (IRS-1) (Zheng and Wang 2021). Consequently, dysregulation of brain insulin signaling is implicated in the development of neurodegeneration, cognitive impairment, and AD. Findings from preclinical and postmortem studies highlighted that brain insulin signaling is dysregulated in AD mouse and human brains (Griffith et al. 2018). Dysregulation of brain IRS-1 contributes to developing brain insulin resistance (BIR), a hallmark of AD and other neurodegenerative diseases (Craft et al. 2012).

Furthermore, GLP-1 and GIP signaling, which regulate insulin signaling, are dysregulated in AD (Nowell et al. 2023). Recently, activating brain GLP-1/GIP receptors or using analogs of these hormones can reduce AD neuropathology (Nowell et al. 2023). Also, findings from different studies confirmed that TRZ mitigates AD neuropathology (Craft et al. 2019; Talbot 2014). In addition, T2D and obesity are considered potential risk factors in the development of AD (Pugazhenthi et al. 2017). Thus, TRZ may mitigate the cardio metabolic profile in T2D and obesity by alleviating AD neuropathology. However, the underlying neuroprotective mechanism of TRZ against AD is not entirely explained. Hence, this mini-review aims to discuss the probable neuroprotective molecular effect of TRZ in the management of AD.

## Pathogenesis of AD

AD is the most common neurodegenerative disease and represents the most typical cause of dementia (Al-Kuraishy et al. 2023a, b, c, d, e, f). AD is caused by the progressive accumulation of extracellular amyloid beta (Aβ) peptide and intracellular neurofibrillary tangles (NFTs) (Al-Kuraishy et al. 2023a, b, c, d, e, f; Alrouji et al. 2024). Aβ is produced from amyloid precursor protein (APP) by α, β and γ secretase that regulates neurotransmitter release from presynaptic neurons (Ali et al. 2024a, b). APP is processed by two main pathways including amyloidogenic and non-amyloidogenic pathways. In the amyloidogenic pathway, APP via β and γ secretases is converted to the neurotoxic Aβ which promote the formation of amyloid plaque. However, in the non-amyloidogenic pathway, APP via α secretase is converted to the soluble APP alpha (sAPP α) which has a neuroprotective effect by reducing the accumulation of neurotoxic Aβ and the formation of amyloid plaque (Pradeepkiran et al. 2019). Furthermore, Aβ aggregates increase the hyperphosphorylation of tau by mediating the activation of cyclin-dependent kinase 5 (CDK5). Caspases are cysteine aspartate proteases that directly cleaved APP during apoptosis and lead to the formation of elevated Aβ. An increase in the interaction between tau protein and Aβ was found to be associated with a damaged neuronal condition that could cause cognitive deficits in AD patients (Pradeepkiran et al. 2019). Interestingly, γ-secretase is mainly mediating the interaction between Aβ and tau protein. It has been shown that γ-secretase modulators such as ibuprofen inhibit tau protein hyperphosphorylation (Lanzillotta et al. 2011).

Importantly, Aβ is eliminated from the brain across the blood-brain barrier (BBB) into the systemic circulation when it metabolized by the liver and excreted by kidney (Al-Kuraishy et al. 2023a, b, c, d, e, f, 2024a, b; Alsubaie et al. 2022; Ali et al. 2024a, b). In addition, Aβ is eliminated by cellular proteostasis and neuronal autophagy (Barmaki et al. 2023). Furthermore, Aβ is degraded by brain enzymes neprilysin (NEP) and insulin-degrading enzyme (IDE) (Kato et al. 2022). In this state, mutations of the *APP* gene augment the assembly of aberrant insoluble Aβ, leading to the formation of amyloid plaque (Tomiyama and Shimada 2020). Moreover, dysregulation of tau protein phosphorylation, which regulates axonal transport and neuronal stability, is implicated in the pathogenesis of AD by inducing the formation of NFTs (Tomiyama and Shimada 2020).

Of note, genetic factors such as mutations in *APP* and presenilin-1 (*PSN-1*) genes are intricate in the development of early-onset AD, also called familial AD (Ayodele et al. 2021). However, sporadic AD, which forms 95% of AD, is caused by environmental factors and is involved in developing late-onset AD (Arnsten et al. 2021). Mainly, familial AD is caused by the overproduction of Aβ (Ayodele et al. 2021), though sporadic AD is caused by defective clearance of Aβ (Arnsten et al. 2021).

**Fig. 2:**
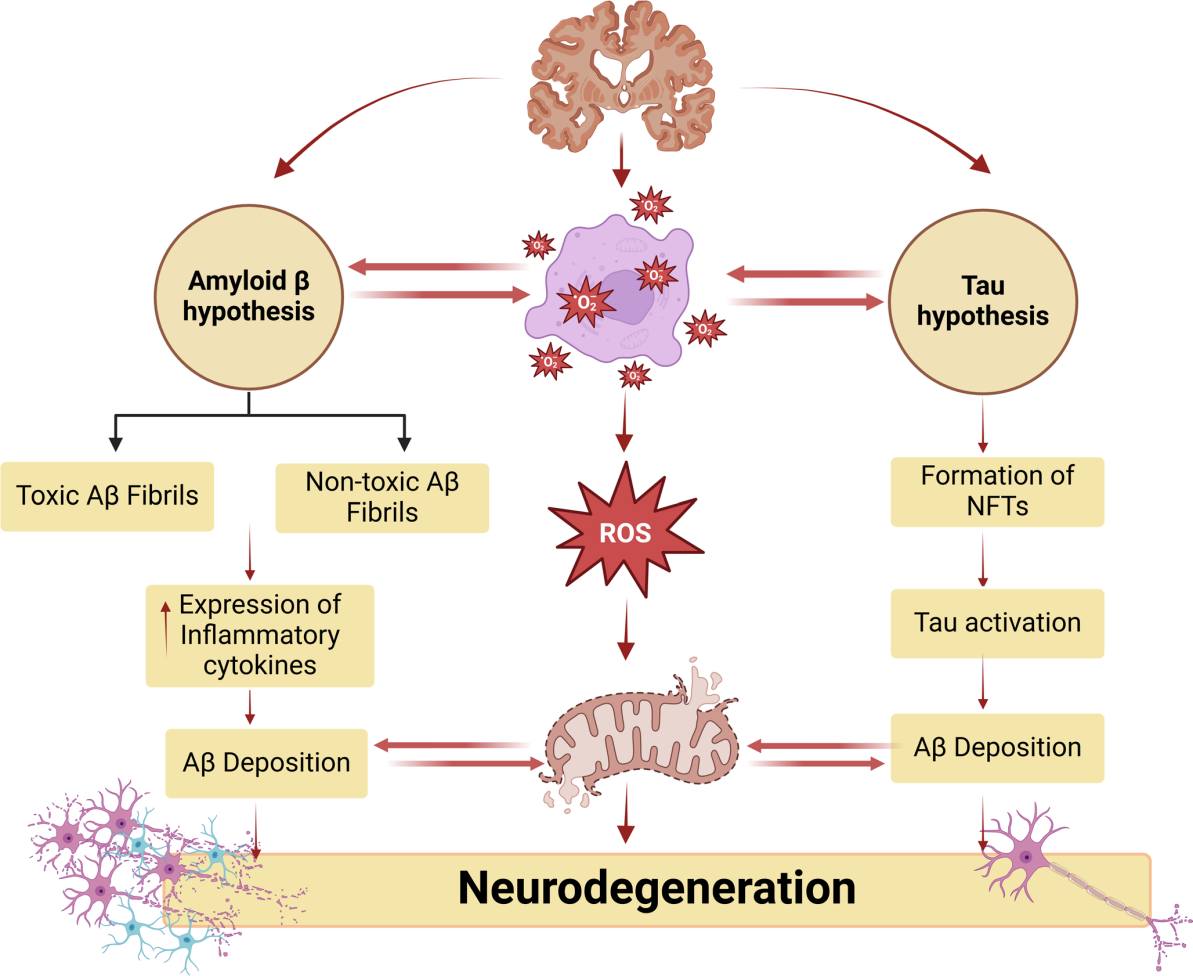
Pathophysiology of AD

These neuropathological changes initiate microglia activation, neuroinflammation, progressive neuronal apoptosis, synaptic dysfunction, impairment of cholinergic neurotransmission, synaptic dysfunction, and the development of cognitive decline in AD (Chen et al. 2022) (Fig. 2).

### The neuroprotective effect of TRZ in AD

Neuroinflammation and oxidative stress are the potential hallmarks of AD neuropathology. Notably, Aβ-induced neuroinflammation and oxidative stress are induced by activating inflammatory signaling pathways such as NLRP3 inflammasome and nuclear factor kappa B (NF-κB) (Kim et al. 2021; Yang et al. 2024a, b). A preclinical study found that TRZ attenuates high-fat diet-induced cognitive impairment in mice by inhibiting the expression of NLRP3 inflammasome (Ma et al. 2024). Also, intra-peritoneal administration of TRZ inhibits the expression of amyloid plaque and associated neuronal apoptosis in transgenic mice. The regulation of neuronal glucose transporter, neuronal mitochondrial function, and insulin sensitivity in the brain mediates this effect. In addition, TRZ up-regulates many enzymes involved in the improvement of neuronal glucose metabolism such as glucose-6 phosphatase, hexokinase, and phosphofructokinase (Craft et al. 2019). Guo et al. (Guo et al. 2023) confirmed that TRZ mitigates memory and learning impairment by reducing BIR and Aβ accumulation in the hippocampus of diabetic rats. Besides, TRZ improves neurogenesis, neurodegeneration, neuronal apoptosis, and neuronal IR in the SHSY5Y cell line (Fontanella et al. 2024). TRZ has a neuroprotective effect against high-fat diet-induced cognitive impairment in mice. The exact impact of TRZ against the development of cognitive decline is related to the inhibition of microglial activation, neuroinflammation, oxidative stress, and BIR (Ma et al. 2024).

Inversely, findings from a preclinical study illustrated that GLP-1 agonist semaglutide and TRZ did not ameliorate cognitive deficits in transgenic mice (Forny Germano et al. 2024). In addition, neither TRZ nor semaglutide reduced hippocampal amyloid load and associated neurodegeneration in transgenic mice. However, the ideal-induced neurodegeneration and neuroinflammation were not identical to the other AD models. In addition, the short duration of the study (4–5 weeks) and low effective doses of TRZ and semaglutide may limit the neuroprotective effects of these agents in AD models. In addition, this study did not evaluate brain size, rate of neurodegeneration, endoplasmic reticulum (ER) stress, and mitochondrial dysfunction (Forny Germano et al. 2024). Hence, this single experimental study may not be valuable in determining the therapeutic efficacy of TRZ or semaglutide.

These findings emphasized that TRZ can alleviate AD neuropathology either directly by inhibiting Aβ-induced neurodegeneration and associated neuroinflammation and oxidative stress or indirectly by reducing BIR. Moreover, TRZ can act peripherally to mitigate the detrimental effects of T2D and obesity on the development and progression of AD.

### TRZ attenuates obesity-induced AD

Obesity and related cardio-metabolic disorders are associated with the development of AD (Flores-Cordero et al. 2022). In addition, obesity-induced oxidative stress, inflammation, and peripheral IR aggravate the development and progression of AD. It has been shown that adiposity induces the activation of NLRP3 inflammasome, which activates the synthesis and release of pro-inflammatory cytokines such as IL-1β and IL-18 through induction of peripheral IR or by activating neuroinflammatory cascades trigger AD neuropathology (Litwiniuk et al. 2021).

**Fig. 3:**
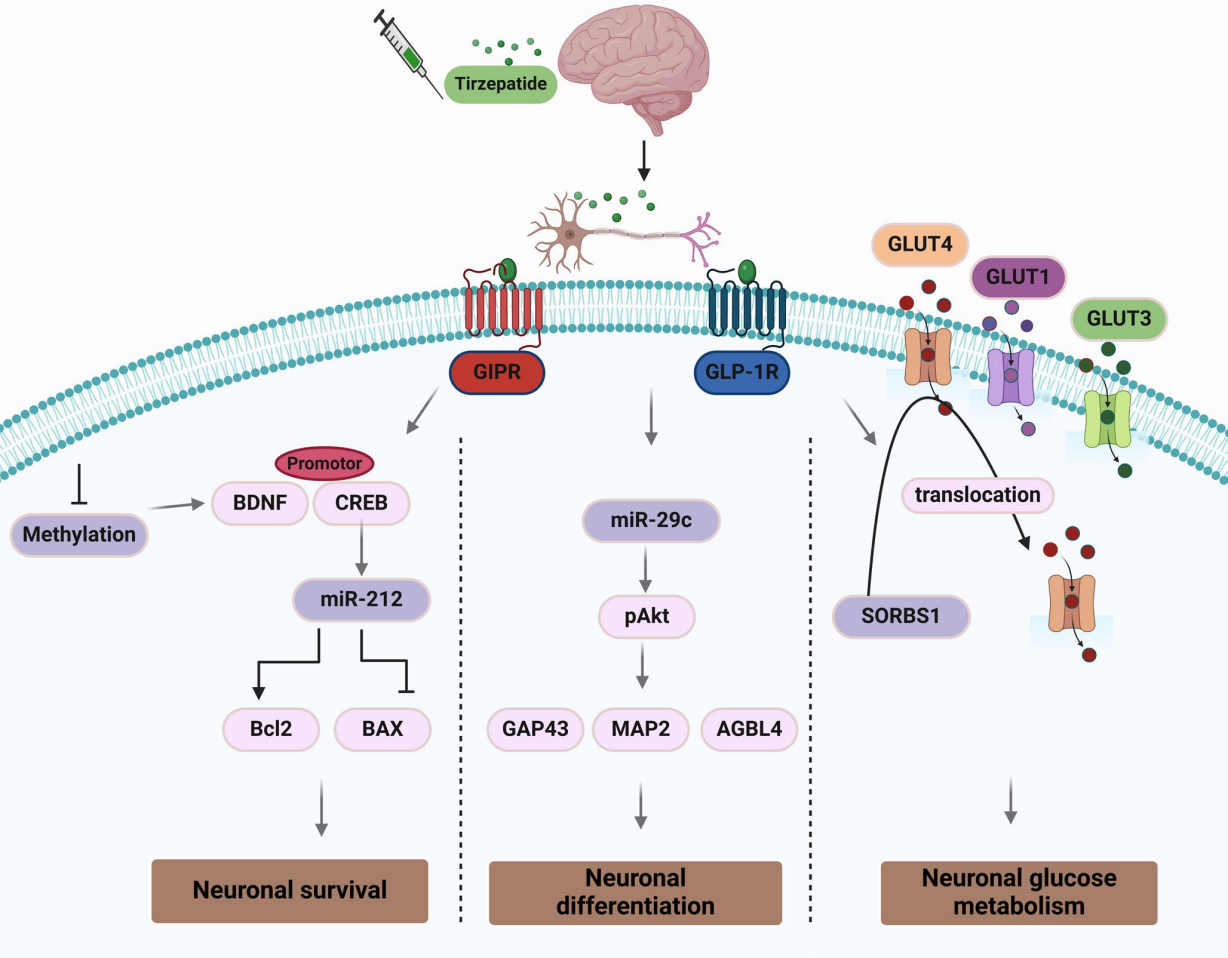
The detrimental effects of obesity in the pathogenesis of AD

Furthermore, dysregulation of adipocytokines, such as leptin, which has a neuroprotective effect against neurodegeneration, is implicated in AD pathogenesis (Bayhaghi 2024). Numerous studies designated that low plasma leptin level was associated with cognitive impairment and AD development (McGuire and Ishii 2016; Albala et al. 2016). It has been revealed that low plasma leptin level is reduced in AD patients compared to healthy controls (Johnston 2014). However, a case-control study found that plasma leptin level was not correlated with cognitive impairment and the severity of AD (Ülker and Kenangil 2018). Consequently, the link between obesity and AD may not be explained concerning the leptin plasma level. Moreover, developing leptin resistance in obesity triggers the dysregulation of hippocampal neurotransmitter release and synaptic plasticity, resulting in AD neuropathology (Thawabteh et al. 2024). Low-grade inflammation due to exaggeration of inflammatory signaling pathways and increasing the release of pro-inflammatory cytokines, mainly IL-1β, IL-6, and TNF-α induce brain leptin resistance in obesity (Paz-Filho et al. 2015; Liu et al. 2015). Because of that, brain leptin resistance could be the potential link between obesity and AD. Direct administration of leptin in AD brains improves hippocampal memory and learning in transgenic mice (McGregor and Harvey 2018). Furthermore, brain leptin resistance promotes Aβ accumulation and tau protein hyper phosphorylation with subsequent deterioration of hippocampal synaptic plasticity (McGregor and Harvey 2018). Maioli et al. (Maioli et al. 2015) illustrated that dysregulation of brain leptin signaling rather than dysregulation of circulating leptin level is implicated in the pathogenesis of AD. Hence, visceral obesity and linked leptin resistance are involved in the pathogenesis of AD (Fig. 3).

TRZ reduces of body weight by 20% and improves lipid profile in obese patients (Chavda et al. 2022). A phase III clinical trial detected that once-weekly TRZ produced a dose-dependent effect in the sustained reduction of body weight of obese patients (Jastreboff et al. 2022). Also, a multicenter, double-blind clinical trial demonstrated that once-weekly TRZ for 72 weeks at a dose of 10 mg and 15 mg was safe and well-tolerated compared to other incretin-based treatments for weight reduction (Jastreboff et al. 2022). A systematic review and meta-analysis revealed that once-weekly TRZ was linked with few adverse effects compared to other anti-obesity drugs (Tan et al. 2023). Moreover, the SURMOUNT-4 clinical trial in 2024 indicated that continued once-weekly TRZ sustained weight reduction in obese patients. However, withdrawal of TRZ therapy leads to a regain of lost body weight (Aronne et al. 2024). Furthermore, a recent cohort study confirmed that TRZ was more effective than semaglutide for reducing body weight in obese patients (Rodriguez et al. 2024). However, this study did not measure the confounding factors, and the rate of withdrawal due to adverse drug reactions was not evaluated that limit the study’s clinical efficacy.

The fundamental mechanism of TRZ in weight reduction is related to the dual activation of GIP and GLP-1 receptors. Diverse studies highlighted that simultaneous stimulation of GIP and GLP-1 receptors by agonists leads to more weight reduction than activation of either of these receptors in diet-induced obesity (Baggio and Drucker 2021; Finan et al. 2013). Thus, dual activation of GIP and GLP-1 receptors may have more significant metabolic enhancement effects than activating either. Nevertheless, activation of GIP and GLP-1 receptors did not significantly affect the energy expenditure in obese and diabetic patients (Bergmann et al. 2019, 2020). In addition, preclinical findings highlighted that energy intake in animals treated with TRZ was not considerably different from that treated with semaglutide, signifying that weight reduction mechanism related to TRZ does not differ from that of semaglutide. Hence, other mechanisms are suggested for weight reduction in obesity. TRZ mainly activates GIP receptors more than GLP-1 receptors (Powell and Taylor 2024). Thus, the weight loss mechanism from TRZ may be mediated by the activation of GIP receptors. It has been illustrated that GLP-1 receptors are compromised in obesity (Irwin and Flatt 2009). Interestingly, in chronic hyperglycemia, IR, T2D, and obesity, the GIP signaling is impaired and not able to inhibit appetite and metabolic derangements (Bergmann et al. 2019; Thondam et al. 2017). Hence, restoring GIP signaling by TRZ can reduce body weight and improve the insulin sensitivity in obesity.

Concerning the role of TRZ in reducing the deleterious effects of obesity on the pathogenesis of AD, it has been demonstrated that TRZ can alleviate brain GIP signaling. GIP receptors have neuroprotective effects against AD neuropathology by inhibiting the formation and progression of amyloid plaques and linked neuroinflammation. Therefore, GIP agonists such as TRZ attenuate progressive neurodegeneration in AD and other neurodegenerative diseases (Roh and Choi 2023; Ji et al. 2016). Significantly, activation of GIP receptors alone may induce metabolic disorders (Bailey 2020); hence, dual activation of GIP and GLP-1 receptors by TRZ effectively reduces body weight.

Moreover, similarly, TRZ attenuates oxidative stress and neuroinflammation in obesity (Ma et al. 2024). In addition, TRZ can mitigate brain leptin resistance, which is the possible link between obesity and AD. A preclinical study found that TRZ-inducing weight loss regulates adipocytokines, including adiponectin and leptin, in obese mice (Samms et al. 2021). By inducing the expression of adiponectin, TRZ regulates leptin sensitivity in obese T2D patients and animal models (Roh and Choi 2023; Handy et al. 2011; Rao et al. 2024; Simental-Mendía et al. 2024).

### TRZ attenuates T2D-induced AD

T2D is considered a possible risk factor for the development of AD by inducing BIR and accompanying neuroinflammation and oxidative stress (Sebastião et al. 2014; Craft 2009). It has been shown that T2D and AD share common mechanistic pathways, such as IR, which deteriorate peripheral and central glucose metabolisms. Interestingly, AD is regarded as type 3 diabetes (T3D) due to dysregulation of brain insulin signaling and BIR development, which may be independent of peripheral IR (Sebastião et al. 2014; Nguyen et al. 2020; Craft et al. 2013; Schiöth et al. 2012). While both peripheral IR and BIR may involve dysregulated insulin signaling, the two conditions are not identical and not always interrelated. BIR is a distinct entity from that of peripheral insulin resistance. Though both can contribute to cognitive decline in general and contribute to AD, they likely do so through different pathways. BIR can include low levels of brain insulin as well as resistance at the receptor level and therefore would be better described as a deficiency in brain insulin action (Rhea et al. 2022).

**Fig. 4:**
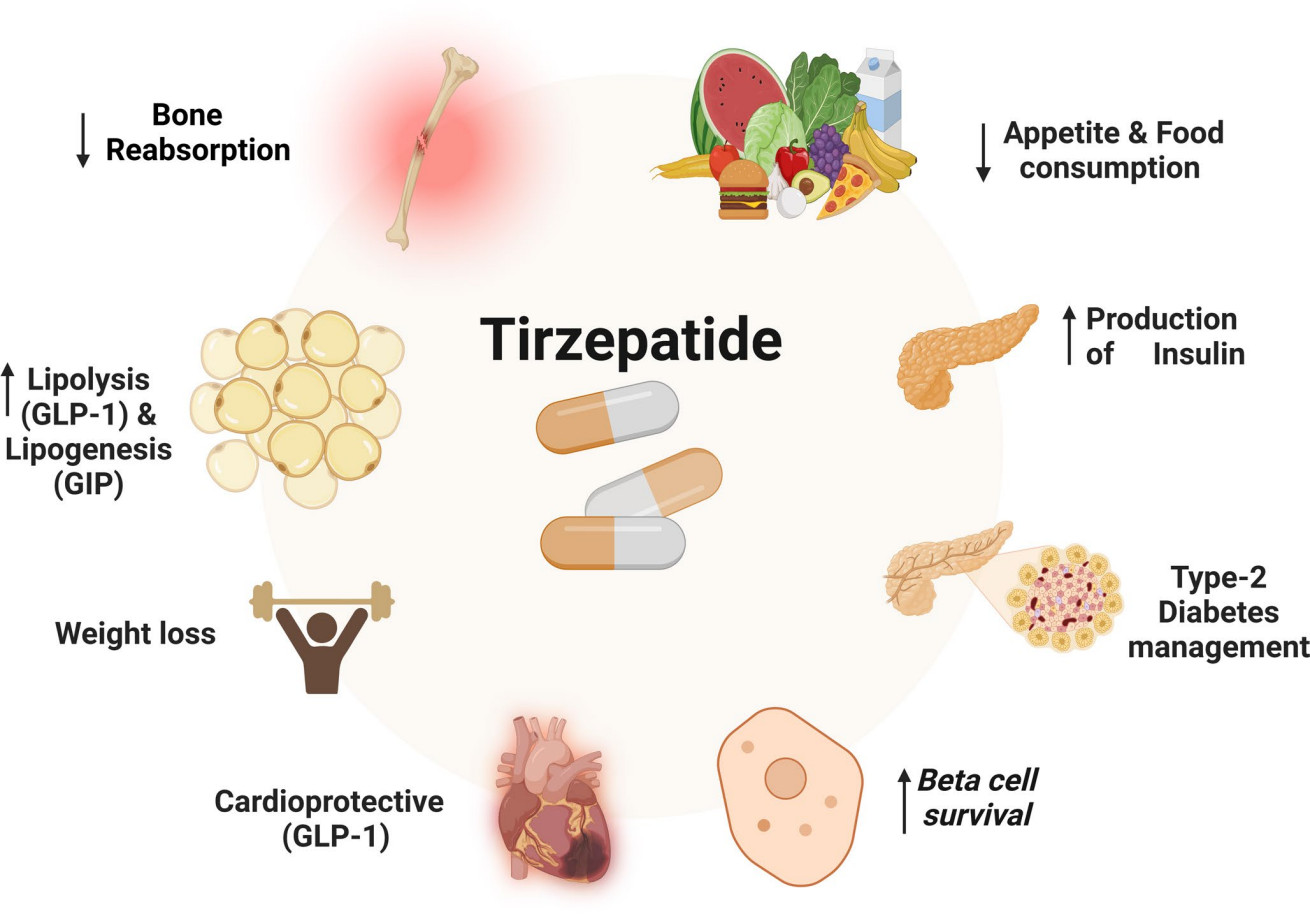
The detrimental effects of T2D in the pathogenesis of AD

It has been suggested that exaggerated the expression of human islet amyloid polypeptide (hIAPP) in T2D is implicated in the pathogenesis of AD. Inhibition of the brain deposition of hIAPP by GLP-1 agonists and dipeptidyl peptidase 4 (DPP-4) inhibitors can reduce the development of AD in T2D (Alrouji et al. 2023). Moreover, low-grade inflammation and oxidative stress in T2D provoke AD development by inducing neuroinflammation and neuronal injury (Barone et al. 2021). Moreover, T2D promotes the generation of neurotoxic advanced glycation end-products, which are implicated in the pathogenesis of AD (Xia et al. 2024). In addition, T2D prohibits neuronal autophagy, which involves the elimination of Aβ, leading to the accumulation of Aβ and other misfolded proteins with subsequent exaggeration of AD neuropathology (Lai et al. 2022; Cui et al. 2021). These verdicts emphasized that T2D can induce the development of AD (Fig. 4).

Hence, targeting peripheral alterations in T2D by TRZ may prevent AD development. A recent preclinical study confirmed that TRZ inhibits adipose tissue inflammation and associated peripheral IR by modulating the ERK signaling pathway and induction of M1-type macrophage apoptosis in obese mouse models (Xia et al. 2024). It has been shown that TRZ inhibits the expression of amyloid plaque and associated neuronal apoptosis in transgenic mice by inhibiting the generation of ROS and the development of brain oxidative stress (Craft et al. 2019). TRZ suppresses oxidative stress and associated inflammation by inhibiting IL-17 signaling in diabetic models (Yang et al. 2024a, b). Hence, TRZ seems to have a neuroprotective effect against T2D-induced oxidative stress and inflammation in AD.

Furthermore, TRZ can reduce the neurotoxic effects of advanced glycation end-products in the induction of the pathogenesis of AD. TRZ attenuates the generation of advanced glycation end-products by ameliorating chronic hyperglycemia in patients with chronic kidney disease (Caruso and Giorgino 2024). TRZ regulates neuronal autophagy, which is highly dysregulated in AD and T2D. Defective autophagy could also link AD and T2D (Wilson et al. 2014). Therefore, activation of cellular autophagy may reduce the pathogenesis of T2D and associated AD neuropathology. Findings from a preclinical study observed that TRZ attenuates diabetic-induced cardiac injury and the development of heart failure by activating cellular autophagy (Taktaz et al. 2024). Similarly, TRZ reduces colistin-induced neurotoxicity by activating neuronal autophagy by modulating PI3 K/AKT/GSK3β in rat models (Hassan et al. 2024). Thus, TRZ, by inhibiting oxidative stress, inflammation, advanced glycation end-products, and activating cellular autophagy, can attenuate the harmful effect of T2D on AD pathogenesis. Taken together, TRZ, by regulating insulin sensitivity and obesity profile, reduces the detrimental effects of T2D and obesity on AD development.

## The molecular mechanism of TRZ in AD

###  Neuronal glucose metabolism

It has been shown that brain neuronal glucose metabolism is highly reduced in AD due to oxidative stress-induced damage of enzymes involved in neuronal glycolysis. Consequently, a reduction in ATP production triggers neurodegeneration, synaptic dysfunction, and the development of cognitive impairment in AD (Butterfield and Halliwell 2019). It has been established that NFTs attenuate the uptake and metabolism of glucose by brain neurons in animal models (Huang et al. 2023). Reduction of neuronal glucose metabolism reduces O-GlcNAcylation, which regulates mitochondrial function and leads to neuronal dysfunction and progression of AD neuropathology (Huang et al. 2023; Dewanjee et al. 2022). Interestingly, brain glucose transporters, mainly GLUT1, GLUT3, and GLUT4, are downregulated and induce progressive neurodegeneration in patients with AD (Dewanjee et al. 2022).

Furthermore, TRZ improves brain neuronal glucose metabolism via activation of the GLP-1 receptor, which upregulates the expression of GLUT1, GLUT3, and GLUT4 by regulating the expression of the *SORB1* gene which regulates insulin signaling (Chang et al. 2018). GLP-1 receptor agonists are effective against AD by reducing neuroinflammation and oxidative stress, neurotrophic effects, decreasing Aβ deposition and tau hyperphosphorylation in AD models (Du et al. 2022). A placebo-controlled, double-blind, phase II clinical trial (the ELAD trial) testing liraglutide in over 200 patients with mild cognitive impairments/AD for 1 year showed that neuronal loss was reduced by the drug (Colin et al. 2023; Edison et al. 2021).

As well, TRZ mitigates BIR by regulating the expression of phosphoinositol triphosphate (PI3 K/AKT) and glycogen synthase kinase three beta (GSK3β) (Guo et al. 2023). It has been established that TRZ inhibits Aβ-induced BIR and associated synaptic dysfunction, memory dysfunction, and cognitive impairment by restoring the PI3 K/AKT/GSK3β signaling pathway in the AD mouse model (Guo et al. 2023). Consistently, many experimental studies revealed that a dual GIP/GLP-1 receptor agonist DA4-JC has a neuroprotective effect against AD neuropathology by regulating the PI3 K/AKT/GSK3β signaling pathway in AD mouse model (Maskery et al. 2020; Cai et al. 2021). PI3 K/AKT is a conserved signaling pathway that regulates signal transduction and many biological processes, such as neuronal proliferation, differentiation, and metabolism. PI3 K/AKT is deregulated in AD due to Aβ and tau protein-induced neurotoxicity (Long et al. 2021). It has been shown that the PI3 K/AKT signaling pathway has a neuroprotective effect against the development and progression of AD (Kumar and Bansal 2022). In addition, PI3 K/AKT is a major regulator of brain insulin signaling and controls the functional activity of astrocytes and microglia (Gabbouj et al. 2019). Therefore, deregulation of brain PI3 K/AKT is linked with the development of BIR and neuroinflammation and progression of AD neuropathology.

Furthermore, GSK3β is a multi-functional enzyme that regulates neurodevelopmental processes and synaptic function. However, mutation of genes involved in the expression of GSK3β and exaggerated signaling provokes the development and progression of AD by inducing the production and the accumulation of Aβ and tau protein (Chauhan et al. 2022; Shri et al. 2023). Additionally, GSK3β inhibitors remarkably manage AD by reducing neurodegeneration, neuroinflammation, and oxidative stress (Shri et al. 2023). Furthermore, AD exaggerates neuronal GSK3β signaling pathway which is implicated in developing BIR by distorting neuronal insulin signaling. Deleting of neuronal GSK3β restores brain insulin signaling and improves neuronal energy homeostasis in the AD rat model (Gupta et al. 2021). Therefore, restoring the balance of the PI3 K/AKT/GSK3β signaling pathway by TRZ improves neuronal glucose metabolism in AD.

### Neuronal survival and neurogenesis

Neuronal survival is reduced in AD due to the induction of neuronal apoptosis, necrosis, ferroptosis, and pyroptosis by Aβ and tau protein, and associated neuroinflammation, oxidative stress, ER stress, and mitochondrial dysfunction (Mangalmurti and Lukens 2022; Shim et al. 2021; Mohamed and Bars 2024). Interestingly, deficiency of telomerase reverse transcriptase (TERT), which enhances neuronal survival, is highly reduced in AD and associated with Aβ-induced neurodegeneration (Shim et al. 2021). In addition, adult hippocampal neurogenesis that persists through life is highly reduced and contributes to cognitive dysfunction in AD. Adult hippocampal neurogenesis is responsible for brain development in neonates and synaptic plasticity in adult life (Choi and Tanzi 2023). It has been shown that adult hippocampal neurogenesis sharply declined in the early stage of AD neuropathology (Belsham et al. 2009). Reduction of adult hippocampal neurogenesis is correlated with cognitive impairment in AD patients (Yassa et al. 2010). The underlying molecular mechanisms for the impairment of adult hippocampal neurogenesis and neuronal survival in AD are related to reduction the expression and release of brain-derived neurotrophic factor (BDNF) (Bhattarai et al. 2020). BDNF has a neuroprotective effect against the development and progression of AD by enhancing neuronal survival, neurogenesis, synaptic plasticity, and remyelination of neurons by activating the proliferation and differentiation of neural stem cells (Zota et al. 2024). BDNF also improves the expression of cAMP-response element-binding protein (CREB), which enhances neuronal survival and neurogenesis. CREB is a ubiquitous transcription factor that regulates neuronal survival, neurogenesis, neuronal differentiation, synaptic plasticity, memory, and cognitive functions. Impairment of CREB signaling is linked with the development of neurodegenerative diseases, including AD (Sharma and Singh 2020). CREB controls neuronal survival by modulating the expression of miR-212-3p, which controls apoptotic and anti-apoptotic pathways. Findings from the preclinical study confirmed that miR-212-3p has a neuroprotective effect against the pathogenesis of AD by inhibiting the expression of NLRP3 inflammasome, attenuating the expression of β-secretase and reducing neuronal pyroptosis in AD rat model (Nong et al. 2022). However, aberrant expression of miR-43a-5p by inhibiting CREB signaling is implicated in developing AD neuropathology in AD patients (Cosín-Tomás et al. 2017). Therefore, activation of brain BDNF signaling and regulating downstream and associated signaling could be a potential therapeutic strategy in managing AD.

**Fig. 5:**
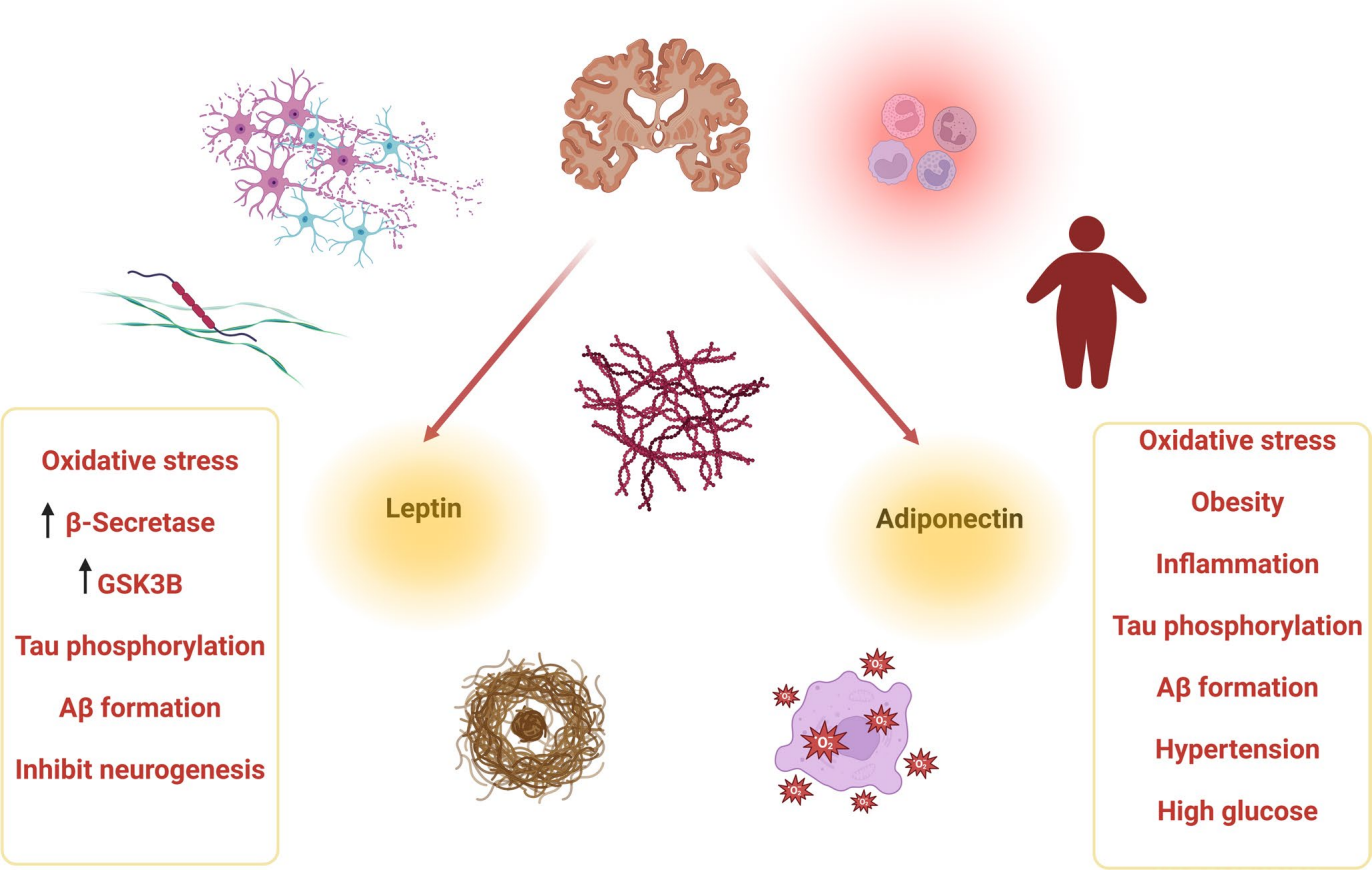
The molecular mechanism of TRZ in AD

It has been shown that TRZ improves neuronal survival in different neurodegenerative diseases, including AD, by activating brain GIP receptors, which enhance transduction pathways involved in neural growth (Talbot 2014). In addition, TRZ alleviates cognitive impairment and memory deficits in animal models by activating hippocampal neurogenesis (Guo et al. 2023). Findings from the preclinical study indicated that TRZ attenuates high-fat diet-induced cognitive impairment through modulation of different signaling pathways, including BDNF (Ma et al. 2024). Likewise, TRZ, through modulation of GIP/GLP-1 signaling, promotes CREB signaling (Mayendraraj et al. 2022). Furthermore, TRZ regulates neuronal survival and apoptosis by regulating the expression of miR-212-3p and miR-43a-5p (Fontanella et al. 2024). However, TRZ, by activating GIP/GLP-1 signaling, promotes the expression of neuroprotective miR-29c-5p, which modulates Aβ production and associated neurotoxicity (Wohlers et al. 2020). In addition, the activated miR-29c-5p via induction of AKT signaling promotes the expression of growth-associated phosphoprotein 43 (GAP-43), improving neuronal growth and synaptic plasticity (Holahan 2017). *GAP-43* gene expression is necessary for structural remodeling of synapses. It has been shown that the downregulation of GAP-43 precedes the onset of AD neuropathology, signifying that GAP-43 has a neuroprotective effect against the pathogenesis of AD (Perovic et al. 2024). In addition, activated miR-29c-5p promotes the expression of microtubule-associated protein 2 (MAP2) to regulate neurite outgrowth and synaptic function (Kim et al. 2020). Significantly, dysregulation of the expression of the *MAP2* gene may be associated with tauopathy and cognitive impairment in AD (Fray et al. 2022). Furthermore, GLP-1 receptor agonist liraglutide improves the expression of MAP2 and exerts a neuroprotective effect against brain ischemia in animal models (Zhu et al. 2016). GAP-43 and MAP2, via increasing the expression of ATP/GTP binding protein-like 4 (AGBL4), promote neuronal differentiation of cholinergic neurons in AD (Baskerville et al. 2008). Therefore, indirect activation of GAP-43, MAP2, and AGBL4 by TRZ can improve neuronal differentiation in AD (Fig. 5).

Taken together, TRZ attenuates the detrimental effects of T2D and obesity on the development and progression of AD by mitigating chronic hyperglycemia in T2D and reducing body weight in obesity. TRZ acts peripherally to attenuate the central effects of low-grade inflammation and oxidative stress in T2D and obesity. In addition, TRZ acts centrally by attenuating the development and progression of AD neuropathology by inhibiting neuroinflammation and brain oxidative stress. TRZ regulates and restores different signaling pathways such as PI3 K/AKT, GSK3β, BDNF, CREB signaling, miR-212-3p, and miR-43a-5p that are involved in the regulation of neuronal survival, growth, and differentiation in AD. Despite of these findings, most findings regarding the neuroprotective effects of TRZ are obtained from preclinical studies that have not been completely translated into clinical settings. Therefore, future clinical studies are recommended in this regard.

## Conclusions

TRZ is a dual agonist of GLP-1 and GIP receptors that was recently approved for treating T2D and obesity. T2D and obesity, by inducing peripheral low-grade inflammation and oxidative stress, aggravate the development of central neuroinflammation and oxidative stress. T2D and obesity are regarded as potential risk factors involved in the development and progression of AD. TRZ, by inhibiting oxidative stress, inflammation, advanced glycation end-products, and activating cellular autophagy, can attenuate the harmful effect of T2D on AD pathogenesis. TRZ can mitigate brain leptin resistance, which is the possible link between obesity and AD. TRZ also induces adiponectin expression, and TRZ regulates leptin sensitivity in T2D patients with obesity. Thus, TRZ, by regulating insulin sensitivity and obesity profile, reduces the detrimental effects of T2D and obesity on the pathogenesis of AD.

Collectively, TRZ attenuates the detrimental effects of T2D and obesity on the development and progression of AD by mitigating chronic hyperglycemia in T2D and reducing body weight in obesity. TRZ acts peripherally to attenuate the central effects of low-grade inflammation and oxidative stress in T2D and obesity. TRZ acts centrally by attenuating the development and progression of AD neuropathology by inhibiting neuroinflammation and brain oxidative stress. TRZ regulates and restores different signaling pathways such as PI3 K/AKT, GSK3β, BDNF, CREB signaling, miR-212-3p, and miR-43a-5p that are involved in the regulation of neuronal survival, growth, and differentiation in AD. Despite of these findings, most findings regarding the neuroprotective effects of TRZ are gained from preclinical studies that are not entirely translated into clinical settings. Accordingly, future clinical trials and clinical studies are suggested in this concern.

## Data Availability

No datasets were generated or analysed during the current study.
